# Polypharmacy and potentially inappropriate medications among hospitalized older adults with COVID-19 in Malaysian tertiary hospitals

**DOI:** 10.1186/s40545-022-00504-1

**Published:** 2023-01-12

**Authors:** Chee-Tao Chang, Siti Mallissa Mohd Shariff, Nur Suriana Abu Bakar, Nasiha Sufina Ramzuzzaman, Chun Kiat Lim, Eddy Yew Joe Lim, Peng Seng Ong, Jie Min Lee, Aie Yen Tan, Siti Fatimah Kamis, Wei Mun Liew, Yuet Man Low, Doris George, James Yau Hon Voo, Hoo Seng Tan, Philip Rajan, Shaun Wen Huey Lee

**Affiliations:** 1 Clinical Research Centre (CRC) HRPB Ipoh, Hospital Raja Permaisuri Bainun, Ministry of Health Malaysia, Ipoh, Malaysia; 2 School of Pharmacy, Monash University Malaysia, Subang Jaya, Malaysia; 3 Pharmacy Department, Hospital Sungai Buloh, Ministry of Health Malaysia, Sungai Buloh, Malaysia; 4 Pharmacy Department, Hospital Pulau Pinang, Ministry of Health Malaysia, George Town, Malaysia; 5 Pharmacy Department, Hospital Sultan Ismail, Ministry of Health Malaysia, Johor Bahru, Malaysia; 6 Pharmacy Department, Hospital Raja Permaisuri Bainun, Ministry of Health Malaysia, Ipoh, Malaysia; 7 Pharmacy Department, Hospital Duchess of Kent, Ministry of Health Malaysia, Sandakan, Malaysia; 8 School of Medicine, Faculty of Health and Medical Sciences, Taylor’s University, Subang Jaya, Malaysia

**Keywords:** Polypharmacy, Potentially inappropriate medications, Hospital, Older adults, COVID-19, Malaysia

## Abstract

**Introduction:**

Older adults are among the most vulnerable groups during the COVID-19 epidemic, contributing to a large proportion of COVID-19-related death. Medication review and reconciliation by pharmacist can help reduce the number of potentially inappropriate medications but these services were halted during COVID-19.

**Aim:**

To assess the prevalence and factors associated with inappropriate medicine use among older populations with COVID-19.

**Methods:**

This was a cross-sectional, retrospective analysis of medications among hospitalized older adults with COVID-19. Potentially inappropriate medication use was categorized using the Beer’s and STOPP criteria.

**Results:**

Combining both criteria, 181 (32.7%) of the 553 patients were identified to have used at least one or more potentially inappropriate medication. A marginally higher number of inappropriate medications was documented using the Beers 2019 criteria (151 PIM in 124 patients) compared to STOPP criteria (133 PIMS in 104 patients). The long-term use of proton pump inhibitors (*n* = 68; 12.3%) and drugs which increases the risk of postural hypotension were the most commonly reported PIM (*n* = 41; 7.4%). Potentially inappropriate medication use was associated with previous history of hospital admission in the past 12 months (Odds ratio [OR]: 2.27; 95% CI 1.29–3.99) and higher number of discharge medications.

**Conclusions:**

Nearly, one in three older adults with COVID-19 had been prescribed a PIM, and the proportion of older adults with polypharmacy increased after discharge. This highlights the importance of having clinical pharmacist conducting medication reviews to identify PIMs and ensure medication appropriateness.

**Supplementary Information:**

The online version contains supplementary material available at 10.1186/s40545-022-00504-1.

## Introduction

Globally, the world is getting older. People are expected to live longer and this transcends across all countries with an increase in proportion and size of older adults in the population. As people age, there is a gradual decrease in physical and mental capacity, and they are more likely to experience several conditions at the same time. As a result, they tend to take multiple medications daily, diseases, a condition termed polypharmacy [1]. Polypharmacy is of concern to older adults, as it has been associated with greater risk of adverse outcomes, and leads to an increase of hospitalization and substantial increase in healthcare costs [2].

Epidemiological studies have suggested that polypharmacy is associated with potentially inappropriate medications (PIM), where the risk of using of such medications outweighed their benefits [3]. To identify PIMs, several assessment tools have been developed, the most common being the Beers' criteria, the Screening Tool of Older Persons' Potentially Inappropriate Prescriptions (STOPP), and the Potentially Inappropriate Medications In The Elderly (PRISCUS) list [4–6].

Studies performed to date have shown that the prevalence of reported PIMs varies, depending on the setting, tool used and population examined. In Malaysia, reported prevalence of PIM ranged from 13.6% in a primary care setting [7] to 34.9% in a hospitalized population [8]. Another study in Malaysia, meanwhile, only reported a prevalence of 21.4% among those in the nursing homes [9]. Reasons for these vary, but include the variability in care provision, settings as well as population examined.

In Malaysia, the health care system is supported through a tax funded public system which co-exists with a thriving private sector that is funded mainly through out-of-pocket expenses [10]. The public sector currently serves between 60% and 70% of the population, with the remaining seeking care in the private sector. In 2020, as part of the government’s effort to contain the COVID-19 outbreak, all patients diagnosed with COVID-19 were required to be hospitalized in public hospitals [11]. This provided us with a unique opportunity to assess the prevalence and types of PIM among older adults hospitalized in Malaysia. In this study, we aimed to assess the prevalence and types of PIMs, polypharmacy, and the associated factors among older adults with COVID-19 in Malaysia.

## Methods

This was a cross-sectional, retrospective analysis of admission and discharge medications among hospitalized older adults with COVID-19 in five hospitals across Malaysia in the states of Penang, Selangor, Sabah, Perak and Johor. All hospitals were the tertiary referral centres, where patients diagnosed with COVID-19 were hospitalized as part of the national action plan to contain the spread of COVID-19. At each participating hospital, the clinical pharmacist identified older patients aged 60 years and above who were admitted between October 2021 and July 2022. Moribund patients, those who opted to discharge at own risk or patients who died during their hospital stay were excluded. Patients with a second admission due to COVID-19 were also excluded. Based on power calculations (Raosoft Seattle), a sample size of 311 patients was required assuming a point prevalence of 29%. As the study only analyzed data from medical records, individual consent was not obtained. The study was approved by the Malaysian Research and Ethics Committee (MREC: KKM/NIH/P21-1725 (3)).

### Criteria for potentially inappropriate medication

To determine the use of potentially inappropriate medications, we used all explicit criteria previously published based upon the Beer’s and STOPP criteria, using them separately and combined. We considered all inappropriate medication, where definition of inappropriateness was limited to long-term use that we could not ascertain. Only systematically acting formulations were analyzed. Investigators documented all relevant information including sociodemographics characteristics (e.g., age, gender), COVID-19 categories and medications during admission and discharge medications.

### Data collection

Data were collected from the medical records at the time of admission, where a comprehensive list of medications is routinely compiled by the pharmacist as part of admission procedure. Information on medication at discharge was retrieved from the discharge prescription issued by the attending physician, which includes all regular and as needed medications.

### Statistical analyses

Data were analyzed using IBM SPSS Statistics for Windows version 27 (IBM Corporation, NY, USA). Univariate logistics regression was performed to determine the predicting factors of PIMs, expressed in adjusted odds ratio and 95% confidence interval. A multivariate logistic regression model was created to determine patient-related characteristic associated with PIM use. A two-tailed *p* < 0.05 was considered as the level of statistical significance. The study adhered to the Strengthening Reporting of Observational Studies in Epidemiology (STROBE) guidelines.

## Results

A total of 553 patients were included in this study, and were aged between 60 and 98 years old (median age: 67 [IQR 64–74] years); 300 were females (54.2%); who were hospitalized between 1 and 175 days (median, 8.00 [IQR 5.00–12.00]) and they had been taking 0 to 19 (median, 5.00 [IQR 3.00–7.00]) medications. Most of the patients were hospitalized with Category 3 or 4 COVID-19 (67.5%), with 43 requiring ICU admission (7.8%) and 49 required the use of mechanical ventilation during hospitalisation (8.9%). More than half of the patients have polypharmacy (5 or more medications) during admission (280, 50.6%) and discharge (346, 62.5%). Further details of the patients are shown in Table 1 and Additional file 1: Table S1.

**Table 1:** Demographic characteristics, number of medications and PIMs detected among older adults studied (*n* = 553)

Characteristics	No (%)
Age, median (IQR) years	67 (64–67)
60–70	363 (65.6)
71–80	139 (25.1)
81 and above	51 (9.2)
Hospital admission days, median (IQR) days	8 (5–12)
Gender	
Male	253 (45.8)
Female	300 (54.2)
Presence of comorbidities	
No	231 (41.8)
Yes	322 (58.2)
Hospitalization in the past 12 months	
No	487 (88.1)
Yes	67 (11.9)
COVID-19 severity during admission	
Category 1	58 (10.5)
Category 2	82 (14.8)
Category 3	133 (24.1)
Category 4	240 (43.4)
Category 5	25 (4.5)
NA	15 (2.7)
ICU admission	
No	510 (92.2)
Yes	43 (7.8)
Need for mechanical ventilation	
No	504 (91.1)
Yes	49 (8.9)
Number of medications at admission, median (IQR) drugs	5 (3–7)
Number of medications at discharge, median (IQR) drugs	5 (4–8)
Total number of medications in admission	
0	8 (1.4)
1–4	265 (47.8)
5–10	252 (45.5)
11–15	26 (4.7)
16–20	2 (0.4)
Mean (SD): 5.07 (3.09)	
Total number of medications in discharge	
1–4	207 (37.4)
5–10	309 (55.8)
11–15	36 (6.5)
16–20	0 (0.0)
> 20	1 (0.2)
Mean (SD): 5.79 (2.99)	
Total number of PIMs detected using STOPP	
1	78
2	18
3	4
7	1
Total number of PIMs	133
Mean (SD): 0.25 (0.61)	
Total number of PIMS detected using Beers	
1	102
2	18
3	3
4	1
Total number of PIMs	151
Mean (SD): 0.28 (0.57)	

### Potentially inappropriate medications

Considering both explicit criteria, a total of 181 patients were identified to have used at least one or more potentially inappropriate medication (32.7%). A marginally higher number of inappropriate medications was documented using the Beers 2019 criteria (151 PIM in 124 patients) compared to STOPP criteria (133 PIMS in 104 patients). Table 2 presents the top 10 most commonly used inappropriate medications when all explicit criteria were combined. The long-term use of proton pump inhibitors (*n* = 68; 12.3%) as well as use of drugs which increases the risk of postural hypotension were the most commonly reported PIM (*n* = 41; 7.4%, Additional file 1: Table S2). Multivariate regression showed that hospital admission in the past 12 months and higher number of discharge medications was associated with a higher risk of inappropriate medication use (Table 3).

**Table 2:** Most common potentially inappropriate medication or prescribing identified in 553 older adults who were admitted due to COVID-19

Description	*n* (%)
Prescribed PPIs for at least 8 weeks or without an evidence based clinical indication	68 (12.3)
ACEi, ARB or calcium channel blocker use which increases risk of postural hypotension	41 (7.4)
Prazosin as antihypertensives may cause postural hypotension	16 (2.9)
Hydrochlorothiazide use which can result in deranged blood electrolyte levels	13 (2.4)
Long term use of NSAIDs for > 12 weeks	12 (2.2)
Use of ticlopidine instead of other safer and more effective alternative	7 (1.3)
Beta-blocker may mask hypoglycaemic symptoms in older patients with diabetes	7 (1.3)
Oral elemental iron doses greater than 200 mg daily without evidence of enhanced iron absorption	6 (1.1)
Benzodiazepines use which may increase the risk of falls	6 (1.1)
Loop diuretic without a clinical indication	5 (0.9)

**Table 3:** Binary logistic regressions assessing significant factors associated with presence of potentially inappropriate medications either Beers or STOPP

	Univariate		Multivariate	
	Crude OR/95% CI	*p* value	Adjusted OR/95% CI	*p* value
Age group				
60–70	Reference			
71–80	0.89 (0.58–1.36)	0.582		
81 and above	1.32 (0.72–2.42)	0.363		
Gender				
Male				
Female	1.08 (0.65–1.81)	0.761		
COVID-19 severity				
Category 1	Reference			
Category 2	0.87 (0.40–1.89)	0.715		
Category 3	1.57 (0.789–3.11)	0.200		
Category 4	1.38 (0.72–2.64)	0.329		
Category 5	1.61 (0.59–4.41)	0.352		
Hospital admission in the past 12 months				
No	Reference		Reference	
Yes	2.64 (1.57–4.44)	< 0.001	2.23 (1.29–3.99)	0.004
History of ICU admission				
No	Reference			
Yes	1.38 (0.73–2.61)	0.324		
Mechanical ventilation				
No	Reference			
Yes	1.34 (0.73–2.45)	0.346		
Comorbidities status (at least one comorbid)				
No				
Yes	1.66 (1.14–2.40)	0.008		
Number of discharge medication				
1–4	Reference			
5–10	5.19 (3.25–8.30)	< 0.001	5.33 (3.20–8.58)	< 0.001
More than 10	11.44 (5.24–24.98)	< 0.001	10.75 (4.79–24.16)	< 0.001

CI: confidence interval; OR: odds ratio

Backward stepwise multiple logistic regression analysis was performed. Multicollinearity and interaction term were checked and not found. The Hosmer–Lemeshow test (*P* = 0.927) classification table (overall correctly classified percentage = 71.0%) and area under the curve (70.4%) were applied to check model fitness

## Discussion

To our best knowledge, this was the first and most comprehensive study that assessed the prevalence of PIMs among older adults with COVID-19 using both Beers and STOPP criteria. Similar to existing studies, we noted that more than half of the hospitalized patients had polypharmacy (≥ 5 medications), with around 5% had hyperpolypharmacy, i.e., 10 or more medicines. We also noted that one in three patients were prescribed at least one or more PIMs, the most common being long-term use of proton pump inhibitors and use of antihypertensive agents which increases the risk of postural hypotension.

With a rapidly ageing population worldwide, studies regarding polypharmacy PIMs were extensively conducted in recent years among older adults. A meta-analysis of polypharmacy prevalence among 189,870 patients reported a pooled prevalence of 34.6%, in which most of the studies were conducted in developed European countries [12]. Comparatively, a study in India revealed that the prevalence of polypharmacy among adults admitted in COVID-19 ward was more than 80% [13].

Our study fills a critical evidence gap by quantifying the prevalence of polypharmacy and associated inappropriate prescribing of several common conditions in Malaysia among COVID-19 older patients. While several studies reported the prevalence of polypharmacy and PIMs among Malaysian older adults [2], our study provided insight on the prevalence of polypharmacy and PIMs among COVID-19 older adults as previous studies were conducted among non-COVID-19 individuals [2]. The present study also broadens the evidence on COVID-19 by quantifying the risk of individuals experiencing PIMs and associated outcomes. Similar to our study, Bhardwaj and colleagues reported about one-third of the patients (34.3%) with COVID-19 were prescribed with at least one PIM using the STOPP criteria [14].

We also observed widespread inappropriate use of PPIs, consistent with previous study and contrarily to various guidance on appropriate prescribing, especially in outpatient setting [15]. Given our findings, these results warrant a call to action by key stakeholders for the adoption of medication reviews with particular focus on PPIs especially those who are taking long term. This, however, needs to take into consideration the patient’s preference, given that deprescribing of PPIs may lead to relapse of gastrointestinal symptoms and reduced patients’ satisfaction without clear clinical benefits [16, 17].

Apart from their conventional roles such as clinical rounds and performing medication reviews, pharmacists’ role was expanded greatly during the COVID-19 pandemic. From a global perspective, pharmacists were involved in innovation of COVID-19 risks preventive measures, inventory management, direct patient care and screening, point-of-care antigen testing, and COVID-19 vaccination [18]. In Asian countries, pharmacists were involved in providing free medical and medications counselling, development of COVID-19 practice guidelines, home delivery of medicines, and telemedicines [19]. In Malaysia, pharmacists served as the main pillar of healthcare workforce, involved in managing novel therapies, medication safety follow-up, redesigning medication delivery system, remote medication inventory monitoring, leveraging communication technology in medication counseling in addition to their conventional roles [20–24]. In the context of reviewing medication appropriateness among older adults with COVID-19, implementation of such measures has increased the accessibility and quality of pharmacy services.

There are several limitations in this study. The study was retrospective in nature, and there was no standardized protocol for medication history clerking. Furthermore, protective barriers used by healthcare personnel in this study varied across different settings, and this may affect the quality and standardization of medication history taking during data collection. We did not evaluate the adverse drug events and clinical outcomes of the patients who received the PIMs, and we did not identify the other possible factors associated with potentially inappropriate medicine.

Future studies should consider a larger cohort of patients and long-term follow-up the evaluate PIMs-related adverse events, readmission, falls and mortality.

## Conclusions

In Malaysia, use of potentially inappropriate medication appears to be common. The study underscores the negative effects of inappropriate medication prescribing and are results are critical decision of healthcare stakeholders to implement a medication usage review especially among older adults to reduce any potential harm that could arise from PIMs.

## Supplementary Information

**Additional file 1: Table S1.** Number of medications prescribed based on WHO ATC/DDD index 2022 drug classes during admission and discharge. **Table S2.** Types of PIM detected using STOPP and Beers criteria based on WHO ATC/DDD index during admission and discharge.

## Data Availability

The data sets used in the current study are not publicly available, but are available from the corresponding author on reasonable request.
