# Supplementary material for: Polypharmacy and potentially inappropriate medications among hospitalized older adults with COVID-19 in Malaysian tertiary hospitals
Source: J Pharm Policy Pract. 2023 Jan 12;16:2. doi: 10.1186/s40545-022-00504-1 (PMC9836327; doi:10.1186/s40545-022-00504-1)
Supplement: Supplementary file 1 — Additional file 1: Table S1. Number of medications prescribed based on WHO ATC/DDD index 2022 drug classes during admission and discharge. Table S2. Types of PIM detected using STOPP and Beers criteria based on WHO ATC/DDD index during admission and discharge. [file 40545_2022_504_MOESM1_ESM.docx]

**Table S1: Number of medications prescribed based on WHO ATC/DDD index 2022 drug classes during admission and discharge**

| **Drugs class** | **Admission** | **Discharge** | **Changes (number of item)** |
| --- | --- | --- | --- |
| Drugs for functional gastrointestinal disorders (A03) | 86 | 252 | 166 |
| Drugs for constipation (A06) | 6 | 18 | 12 |
| Antidiarrheals, intestinal antiinflammatory agents (A07) | 1 | 0 | -1 |
| Drug used in diabetes (A10) | 455 | 475 | 20 |
| Vitamins (A11) | 104 | 134 | 30 |
| Mineral supplements (A12) | 70 | 119 | 49 |
| Antithrombotic agents (B01) | 238 | 229 | -9 |
| Antianemic preparations (B03) | 165 | 171 | 6 |
| Cardiac therapy (C01) | 65 | 51 | -14 |
| Antihypertensives (C02) | 24 | 17 | -7 |
| Diuretics (C03) | 109 | 79 | -30 |
| Peripheral vasodilators (C04) | 1 | 1 | 0 |
| Beta blocking agents (C07) | 191 | 167 | -24 |
| Calcium channel blockers (C08) | 313 | 272 | -41 |
| Agents acting on the renin-angiotensin system (C09) | 237 | 159 | -78 |
| Lipid modifying agents (C10) | 371 | 306 | -65 |
| Urologicals (G04) | 33 | 28 | -5 |
| Corticosteroids for systemic use (H02) | 31 | 230 | 199 |
| Thyroid therapy (H03) | 16 | 15 | -1 |
| Antibacterials for systemic use (J01) | 9 | 30 | 21 |
| Antivirals for systemic use (J05) | 10 | 5 | -5 |
| Antineoplastic agents (L01) | 9 | 6 | -3 |
| Immunosuppresants (L04) | 3 | 4 | 1 |
| Antiinflammatory and antirheumatic products (M01) | 1 | 0 | -1 |
| Antigout preparations (M04) | 20 | 16 | -4 |
| Drugs for treatment of bone diseases (M05) | 4 | 3 | -1 |
| Antiepileptics drugs (N03) | 22 | 16 | -6 |
| Anti-parkinson drugs (N04) | 9 | 9 | 0 |
| Psycholeptics (N05) | 8 | 7 | -1 |
| Analgesics (N02) | 11 | 9 | -2 |
| Psychoanaleptics (N06) | 9 | 6 | -3 |
| Drugs used in opiod dependence | 2 | 2 | 0 |
| Drugs for obstructive airway diseases (R03) | 54 | 46 | -8 |
| Cough and cold preparations (R05) | 1 | 7 | 6 |
| Antihistamines for systemic use (R06) | 4 | 8 | 4 |
| Opthalmologicals (S01) | 11 | 8 | -3 |
| Other therapeutic products (V03) | 2 | 2 | 0 |

**Table S2: Types of PIM detected using STOPP and Beers criteria based on WHO ATC/DDD index during admission and discharge**

| **Medications** | **Number of patients** | **Remark** |
| --- | --- | --- |
| **Drugs for functional gastrointestinal disorders (A03)** |  |  |
| Esomeprazole | 5 | Prescribed for more than recommended duration without clear indication |
| Omeprazole | 9 | Prescribed for more than recommended duration without clear indication |
| Pantoprazole | 53 | Prescribed for more than recommended duration without clear indication |
| Rabeprazole | 1 | Prescribed for more than recommended duration without clear indication |
| **Drugs used for diabetes (A10)** |  |  |
| Metformin | 2 | Risk of renal toxicity in pre-existing renal failure (egfr<10) |
| Gliclazide | 3 | Risk of hypoglycaemia |
| **Antithrombotic agents (B01)** |  |  |
| Aspirin | 23 | Patient has uncontrolled hypertension may cause bleeding risk / Not suitable as primary prevention |
| Dabigatran | 4 | Not suitable for impaired renal function (efgr <20) and uncontrolled HTN (1) |
| Rivaroxaban | 1 |  |
| Clopidogrel | 2 | Bleeding risk as patients have uncontrolled hypertension |
| Ticlopidine | 7 | Use alternatives such as clopidogrel or prasugrel with similar efficacy and less side effects |
| Warfarin | 1 | Increased risk of GI bleeding |
| **Antianemic preparations (B03)** |  |  |
| Ferrous fumarate | 6 | Elemental iron doses >200 mg daily do not facilitate absorption |
| Folic acid | 1 | Discontinue as prophylaxis rather than actual treatment of malnutrition |
| Vitamin B1, B6, B12 | 1 | Discontinue as prophylaxis rather than actual treatment of malnutrition |
| Mecobalamin | 1 | Discontinue as prophylaxis rather than actual treatment of malnutrition |
| **Cardiac therapy (C01)** |  |  |
| Digoxin | 1 | Caution in patients with renal failure eGFR<30 |
| Amiodarone | 1 | High risk of side effects compared to alternatives |
| Isosorbide-5-mononitrate | 2 | Increase fall risk |
| **Antihypertensives (C02)** |  |  |
| Methyldopa | 1 | May cause orthostatic hypotension and increase fall risk |
| Moxonidine | 1 | May cause orthostatic hypotension and increase fall risk |
| Prazosin | 16 | May cause orthostatic hypotension and increase fall risk |
| **Diuretics (C03)** |  |  |
| Amiloride/HCTZ | 1 | Risk of hyperkalemia |
| Frusemide | 5 | Safer alternatives available |
| Hydrochlorothiazide (HCTZ) | 13 | May exacerbate pre-existing electrolyte imbalance |
| Spironolactone | 3 | Risk of hyperkalemia |
| Indapamide | 2 | - |
| **Beta blocking agents (C07)** |  |  |
| Bisoprolol | 4 | Risk of heart block in patient with bradycardia/ Possible masking hypoglycemia episode |
| Metoprolol | 3 | Risk of heart block in patient with bradycardia/ Possible masking hypoglycemia episode |
| **Calcium channel blockers (C08)** |  |  |
| Diltiazem | 1 | Risk of worsen heart failure |
| Amlodipine | 21 | Risk of postural hypotension and fall |
| Felodipine | 1 | Risk of postural hypotension and fall |
| **Agents acting on the renin-angiotensin system (C09)** |  |  |
| Perindopril | 16 | Risk of postural hypotension and fall |
| Losartan | 1 | Risk of postural hypotension and fall |
| Telmisartan | 1 | Risk of postural hypotension and fall |
| **Urologicals (G04)** |  |  |
| Terazosin | 4 | Risk of postural hypotension and fall |
| **Corticosteroids for systemic use (H02)** |  |  |
| Prednisolone | 2 | Risk of GI bleed |
| **Antibacterials for systemic use (J01)** |  |  |
| Sulphamethoxazole 400mg/Trimethoprim 80mg | 1 | Risk of hyperkalemia |
| **Antiinflammatory and antirheumatic products (M01)** |  |  |
| Diclofenac | 4 | Risk of GI bleed |
| **Antigout preparations (M04)** |  |  |
| Colchicine | 3 | Risk of colchicine toxicity |
| Escitalopram | 1 | Risk of exacerbate pre-existing hyponatremia |
| **Analgesics (N02)** |  |  |
| Morphine | 1 | Risk of impairing cognitive function and falls |
| **Antiepileptics drugs (N03)** |  |  |
| Carbamazepine | 3 | Risk of impairing cognitive function and falls |
| Gabapentin | 1 | Risk of impairing cognitive function and falls |
| Phenobarbitone | 1 | Risk of impairing cognitive function and falls |
| **Psycholeptics (N05)** |  |  |
| Alprazolam | 2 | Risk of impairing cognitive function and falls |
| Clonazepam | 2 | Risk of impairing cognitive function and falls |
| Lorazepam | 2 | Risk of impairing cognitive function and falls |
| Risperidone | 1 | Risk of impairing cognitive function and falls |
| Quetiapine | 1 | Risk of impairing cognitive function and falls |
| Zolpidem | 1 | Risk of impairing cognitive function and falls |
| **Psychoanaleptics (N06)** |  |  |
| Amitriptyline | 1 | Risk of impairing cognitive function and falls |
| **Drugs for obstructive airway diseases (R03)** |  |  |
| Theophylline | 1 | Risk of toxicity due to narrow therapeutic index |
| **Antihistamines for systemic use (R06)** |  |  |
| Chlorpheniramine | 1 | Risk of impairing cognitive function and falls |
| Diphenhydramine | 2 |  |
| **Other drug class interactions** |  |  |
| Aspirin and diclofenac | 1 | Bleeding risk |
| Aspirin and rivaroxaban | 1 | Bleeding risk |
| Bisoprolol combined with diltiazem | 1 | Increase risk of heart block |
| Atenolol combined with diltiazem | 2 | Increase risk of heart block |
| Spironolactone plus losartan | 1 | Risk of deranged potassium level |
